# An ultra-stable redox-controlled self-assembling polypeptide nanotube for targeted imaging and therapy in cancer

**DOI:** 10.1186/s12951-018-0427-1

**Published:** 2018-12-08

**Authors:** Gitanjali Asampille, Brijesh Kumar Verma, Monalisa Swain, Abhijith Shettar, Steven A. Rosenzweig, Paturu Kondaiah, Hanudatta S. Atreya

**Affiliations:** 1 NMR Research Centre, Indian Institute of Science, Bangalore, 560012 India; 2 Solid State and Structural Chemistry Unit, Indian Institute of Science, Bangalore, 560012 India; 3 Molecular Reproduction, Development and Genetics, Indian Institute of Science, Bangalore, 560012 India; 4 Department of Cell and Molecular Pharmacology, & Experimental Therapeutics, Medical University of South Carolina, Charleston, SC 29425 USA; 5 Present Address: Basic Research Laboratory, National Cancer Institute, Frederick National Laboratory for Cancer Research, Frederick, MD USA; 6 Present Address: Biotechnology Engineering, Ramaiah Institute of Technology, Bangalore, Karnataka 560054 India

**Keywords:** Protein nanotube, Self-assembly, Intermolecular disulfide bonds, RGD motif, Integrin targeting, Cancer

## Abstract

We introduce a self-assembling polypeptide-based nanotube system having the ability to specifically target cancer cells. The nanotubes target the cancer cell surface through integrin engagement with the help of multiple RGD units present along their surface. While the nanotubes are non-toxic towards cells in general, they can be loaded with suitable drugs to be released in a sustained manner in cancer cells. In addition, the nanotubes can be utilized for cellular imaging using any covalently tagged fluorescent dye. They are stable over a wide range of temperature due to intermolecular disulphide bonds formed during the self-assembly process. At the same time, presence of disulphide bonds provides a redox molecular switch for their degradation. Taken together this system provides a unique avenue for multimodal formulation in cancer therapy.

**Electronic supplementary material:**

The online version of this article (10.1186/s12951-018-0427-1) contains supplementary material, which is available to authorized users.

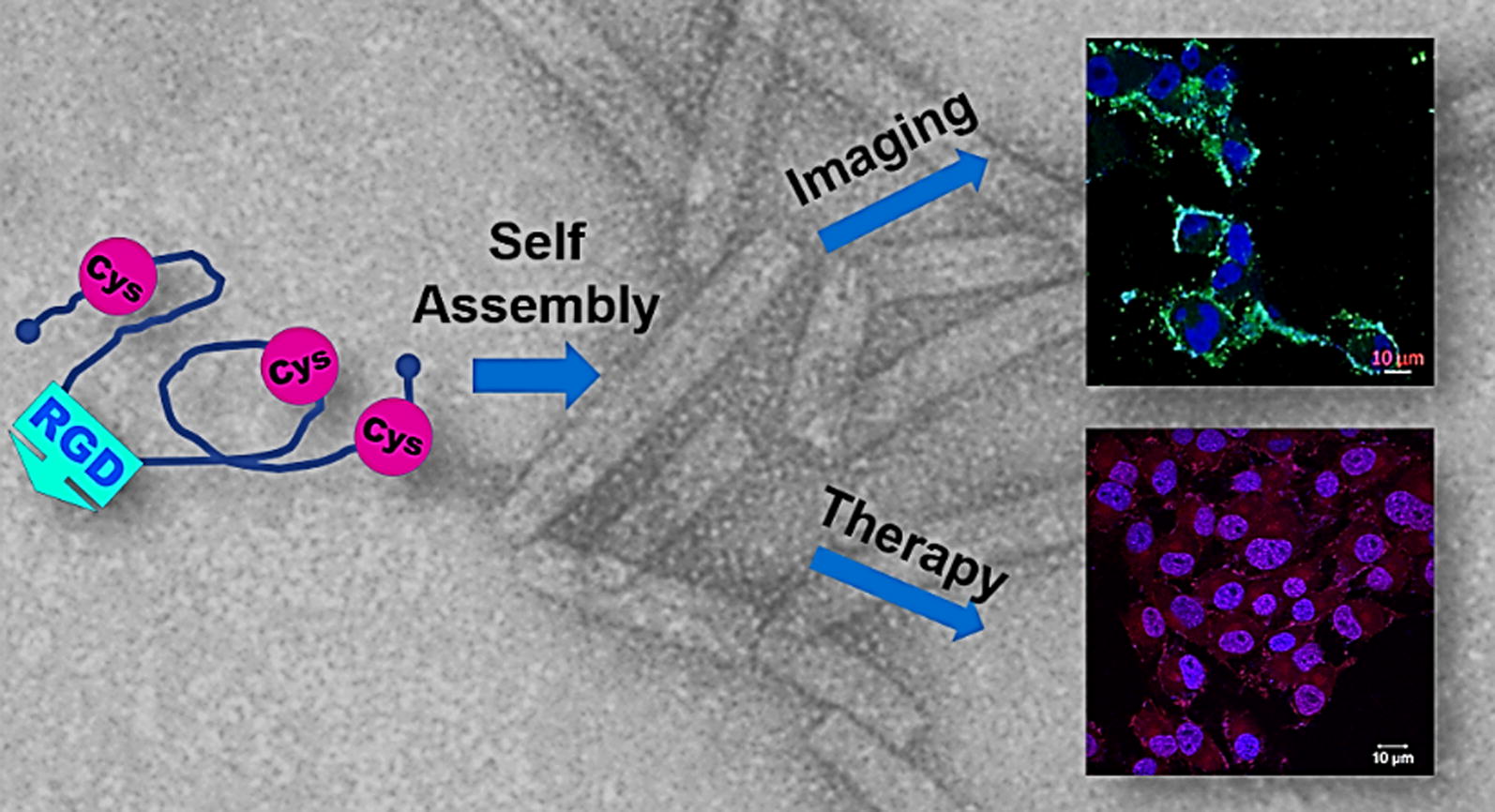

## Introduction

The development of multimodal systems combining imaging and drug delivery components have come into focus due to their theranostic efficacy [1]. Different materials for designing nanocarriers have been proposed such as nanogels [2], polymeric micelles [3], liposomes [4] along with various targeting agents [5]. A desirable property of such systems is the ability to target the tumor cells and release the drug stably in the blood stream [3]. To achieve this, targeted delivery systems have been proposed [6]. The Arg-Gly-Asp (RGD) is the most widely studied and used peptide for decorating biomaterials to achieve specific targeting in the biomedical field [7–12]. This tripeptide has proved to be very effective in binding integrin receptors as efficiently as the principal integrin-binding domains within extracellular matrix (ECM) proteins such as fibronectin, vitronectin and fibrinogen [13]. Integrins are heterodimeric cell surface receptors with alpha and beta subunits [13]. They mediate interaction among cells via their adhesion to the extracellular matrix. There are 24 integrin heterodimers, and several of them are up regulated in various tumor types including breast cancer [14]. The RGD motif exhibits association with several types of integrins such as α5β1, αvβ3 and αvβ5 integrins [14].

Our group had previously introduced nanotubular structures formed by self-assembly of a polypeptide fragment at the C-terminal end (residues 249–289) of human insulin-like growth factor binding protein-2 (hIGFBP-2_249–289_) [15]. Wild type hIGFBP-2_249–289_ has two cysteines in its primary sequence. However, the polypeptide fragment we considered had an additional cysteine due to a mutation at R281 [16]. The polypeptide (hIGFBP-2_249–289_ (R281C)) thus had an odd number of cysteines, which resulted in spontaneous self-assembly to form soluble nanotubular structures via intermolecular disulfide bonds. The nanotubes formation/disassembly can be controlled by choosing suitable redox conditions. Further, the polypeptide fragment contains a RGD motif in its sequence. Upon formation of nanotubes, an array of RGD is displayed on the surface providing a unique feature for active targeting of cancer cells through integrin binding.

We show that the multi-RGD containing protein nanotube activates integrin signalling and can be loaded with a cytotoxic drug. The drug loaded nanotubes show cytotoxicity selectively towards cancer cells and can get dissembled as it approaches the cell environment. In addition, the nanotubes can be used for cellular imaging by conjugating them with a fluorescent tag.

## Results

### Monitoring the self-assembly of the nanotubes

**Fig. 1:**
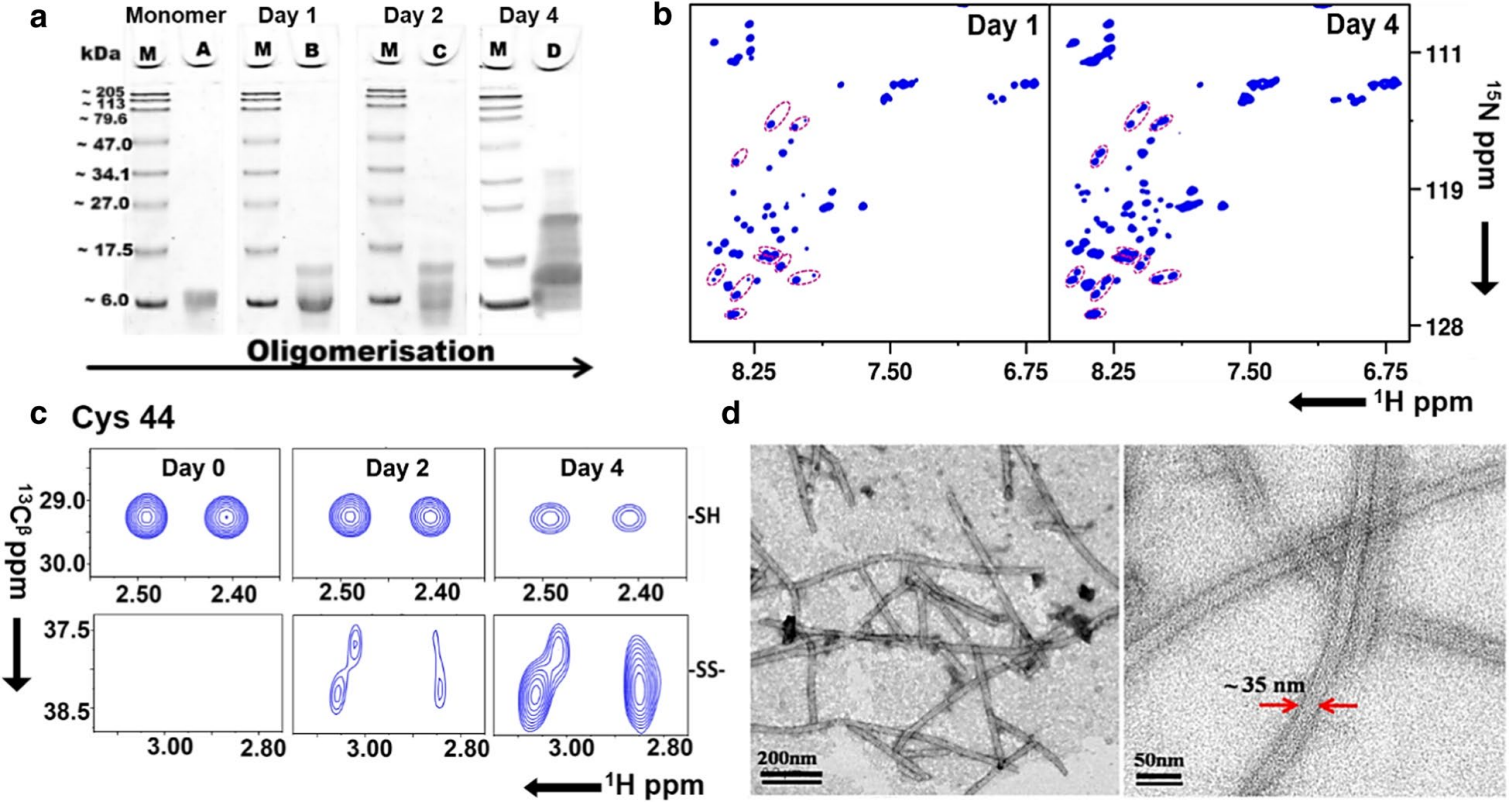
Oligomerization through self-assembly of hIGFBP-2_249–289_ (R281C). **a** SDS-PAGE profile of the oligomerization M: marker, A: Monomer, B: Dimer (day 1), C: more populated dimer (day 2), D: multiple oligomer species (day 4); **b** 2D [^15^N, ^1^H] heteronuclear single quantum correlation (HSQC) spectra on day 1 and day 4, purple color dotted line circles depict the pairs of peaks representing old and new species for some of the amino acid residues; **c** Cys44 was monitored for oxidation of –SH to disulfide (–SS–) with oligomerization, based on ^13^C^β^ shifts observed in the 2D [^13^C–^1^H] HSQC NMR experiment, **d** TEM images of the hIGFBP-2_249–289_ nanotubes

The self-assembly of hIGFBP-2_249–289_ (R281C) was initiated at 298 K in 0.8 mM of the protein dissolved in 50 mM Na-phosphate buffer (pH 6) containing 50 mM NaCl, in the absence of any reducing agent. The oligomerization was tracked in a time-dependent manner using, in parallel, Sodium Dodecyl Sulfate–Polyacrylamide Gel Electrophoresis (SDS-PAGE) (Fig. 1a) and two-dimensional (2D) nuclear magnetic resonance (NMR) spectroscopy (Fig. 1b, c). The observations were made for the total time required to form mature nanotubes i.e. 8 days at room temperature. However, emphasis was on initial time points to characterize intermediates, if any. The presence of oligomeric species was monitored by collecting fractions on Day 1, 2 and 4 and storing them at − 20 °C, which helped in arresting the oligomerization at different time points. The SDS-PAGE profile shown in Fig. 1a depicts the progress of oligomerization during self-assembly at initial stages at room temperature and reveals the appearance of a dimer on day 1, which becomes relatively more populated on day 2 and higher order multimeric species are visible on day 4.

In parallel with SDS-PAGE, at the same time points, the appearance of new molecular species in solution was monitored with the help of 2D [^15^N, ^1^H] heteronuclear single quantum correlation (HSQC) (Fig. 1b), wherein each peak in the 2D spectrum corresponds to one amino acid residue of the protein. Notably, new spectral signatures are observed with the progress in oligomerization. The intensity profiles of a few resonances depicting the decrease in the signal on day 1 and appearance of new resonances as a function of time is shown in Additional file 1: Figure S1. Note that the old resonances represent both old and new conformers for those residues which do not undergo a change in their chemical shift. The low spectral dispersion in the 2D [^15^N, ^1^H] spectrum indicates that the polypeptide fragment is largely unstructured, which was also verified in our earlier studies [15].

The self-assembly of the polypeptide fragment into nanotubular structures is governed by intermolecular disulfide formation as described previously [15]. We therefore examined the oxidation of thiol group of cysteines in the polypeptide as the oligomerization progressed using 2D [^13^C, ^1^H] HSQC NMR experiment. Figure 1c shows an expanded portion of the 2D [^13^C, ^1^H] HSQC spectrum depicting the state of oxidation of one of the three cysteines, Cys 44, that is, the conversion of thiol (–SH) to disulfide (–SS–) due to oxidation. During the course of self-assembly, a significant growth in the cross peak intensity of the resonance corresponding to the disulfide state is seen with the appearance and growth of the peak at ~ 38 ppm, which is a characteristic chemical shift signature of the ^13^C^β^ of cysteine involved in a disulfide bond [17]. At the same time, a reduction in the intensity of thiol peaks at ~ 29 ppm is observed, which is the ^13^C^β^ chemical shift for the reduced thiol group (–SH) of cysteine [17]. The other two cysteines (Cys 12 and Cys 33) were also observed to undergo a similar oxidation profile. After completion, the nanotubes formed were observed using transmission electron microscopy (TEM). The TEM images show the hollow tubular forms (Fig. 1d). The images reveal an outer diameter of ~ 35 nm and an inner diameter of ~ 25 nm.

### Stability of nanotubes

**Fig. 2:**
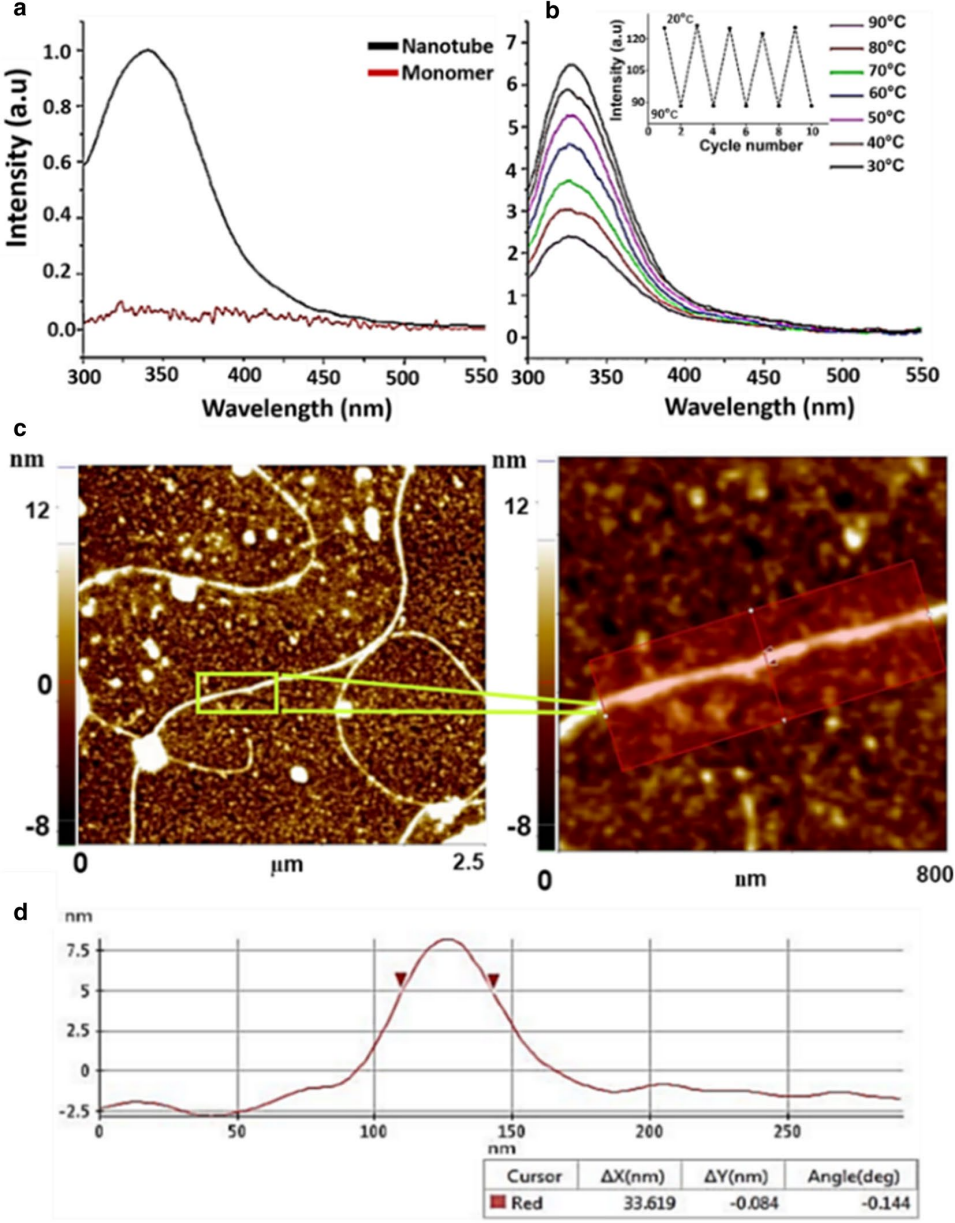
Stability of hIGFBP-2_249–289_ nanotubes. **a** Fluorescence emission spectra of monomer (red) and nanotube (black). **b** Fluorescence emission spectra of hIGFBP-2_249–289_ nanotubes acquired at different temperatures at pH 7.4, inset representing the graph of fluorescence intensities at 20 °C and 90 °C during 10 theromcycles performed. **c** AFM image of the nanotubes, **d** line profile used for calculating the averaged diameter

Fluorescence emission spectra were recorded at various temperatures to probe the stability of nanotubes. The nanotubes have higher intrinsic tyrosine fluorescence compared to the monomer as shown in Fig. 2a, presumably due to the conformational changes in the FY dipeptide motif in the polypeptide chain [15]. The fluorescence spectra were acquired for nanotubes at pH 7 at different temperatures (Fig. 2a). With increases in temperature the intensity of the fluorescence emission spectrum decreases implying quenching of fluorescence with a rise in temperature. However, even at high temperatures (90 °C) the nanotubes are intact, exhibiting significant fluorescence (tyrosine excitation 280 nm) with λ_max_ at 340 nm (Fig. 2a). Figure 2b shows the fluorescence emission spectra at λ_max_ 340 nm for the nanotubes at pH 7. Investigation using atomic force microscopy (AFM) of the mature form of nanotubes that was ~ 9-months old (Fig. 2c), revealed that the morphological characteristics of nanotubes were intact showing a line profile (Fig. 2d) with diameter of ~ 30 nm. Taken together, this implies that these protein nanotubes possess remarkable stability and can be used for various nano-platforms for biomedical applications.

### Effect of nanotube on integrin signalling

**Fig. 3:**
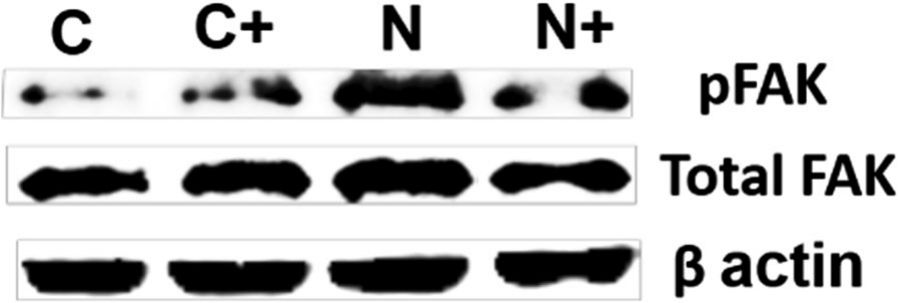
Western blot- showing integrin specificity. C: untreated as control, C+: control in presence of an inhibitor, N: nanotube, N+: nanotube in presence of an inhibitor, inhibitor − RGDS, 1 h treatment followed by 1 h treatment of samples

To probe the accessibility and conformational compatibility of the RGD sites on the nanotube for binding integrins on the cell surface, we used integrin-induced phosphorylation of FAK at tyrosine 397 (pFAKTyr-397) as a measure of integrin engagement and signaling. Interaction of the RGD motif present on the nanotubes with cells via integrin binding leads to an induction of intracellular phosphorylation of FAK. Results shown in Fig. 3 indicates phosphorylation of FAK upon the treatment of HeLa cells with nanotubes (500 ng/ml) for 1 h. Pre-treatment with 25 µM of RGDS peptide, an integrin inhibitor demonstrates the specificity of this interaction. In the absence of inhibitor, the intensity of pFAK was higher compared to control cells. In the presence of the inhibitor, pFAK induction by nanotubes is compromised while control cells do not exhibit significant change. This observation suggests that nanotubes interact with integrins at the cell surface and activates signaling.

### Nanotube-FITC conjugate for imaging cancer cells

**Fig. 4:**
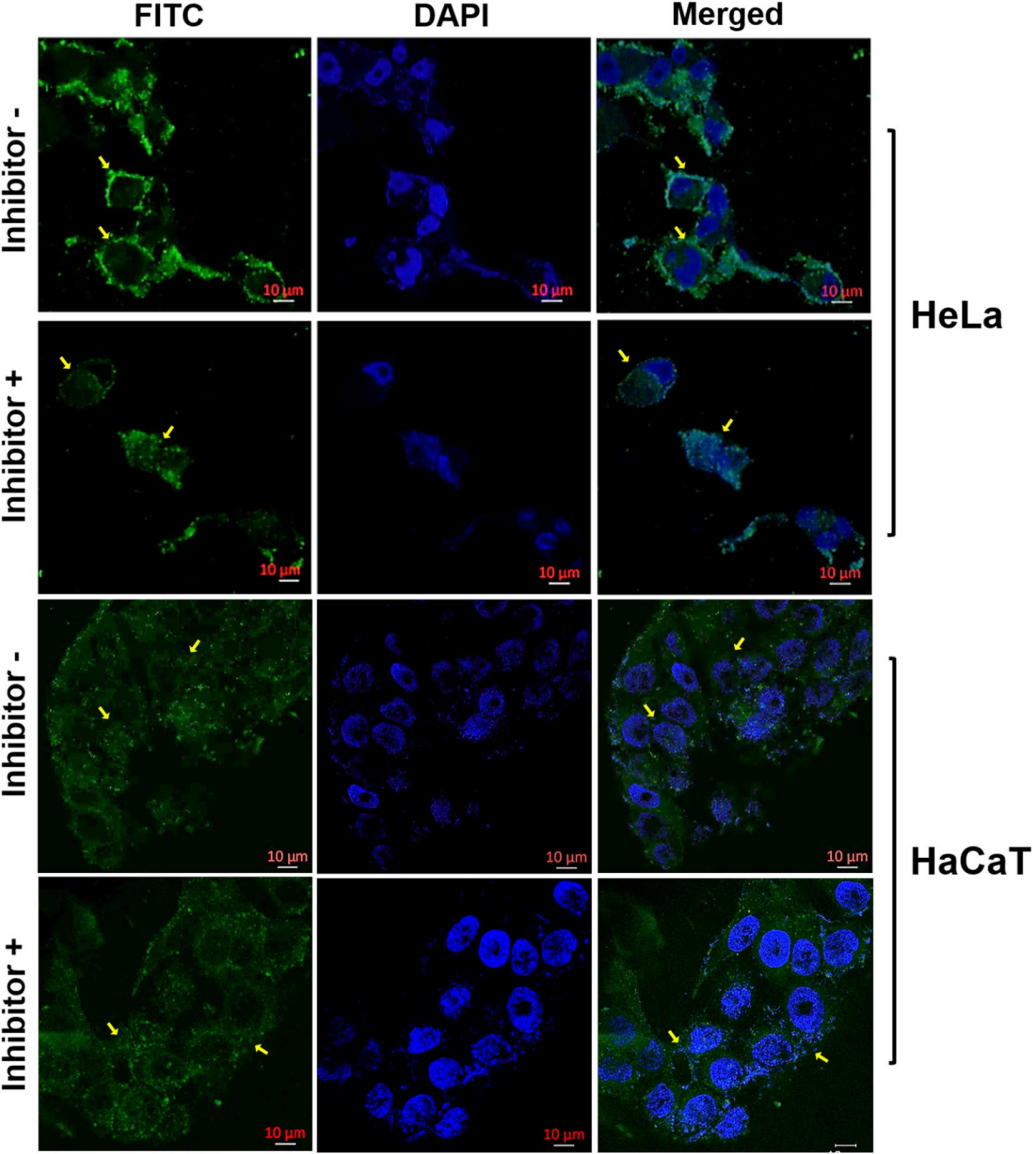
Cancer cell imaging with nanotube-FITC. Cellular interaction of nanotube-FITC with HeLa (top two rows) and in HaCaT (bottom two rows) cells at 4 h (Inhibitor −), Cellular binding of nanotube-FITC following 1 h treatment of the integrin inhibitor given after 4 h of incubation of the conjugate with the cells (Inhibitor +). Cell nuclei are stained with DAPI (blue color) and merged is an overlay of images acquired using FITC and DAPI acquisition channel

**Fig. 5:**
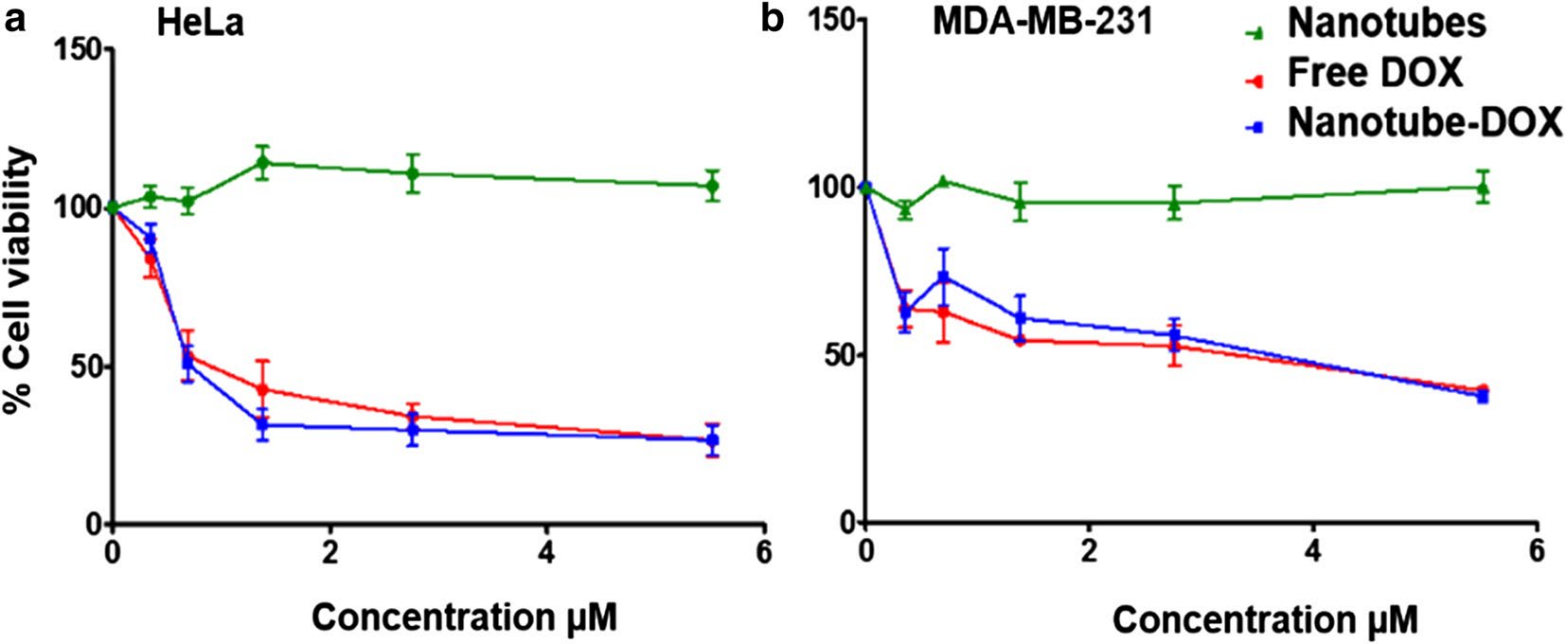
Cytotoxicity evaluation using MTT assay. **a** HeLa, **b** MDAMB231. The IC_50_ of nanotube-DOX for HeLa is 0.69 µM and free DOX is 0.78 µM. In the case of MDAMB231, IC_50_ of nanotube-DOX is 3.45 µM and free DOX is 3.97 µM. suggesting the nanotube-DOX matches the efficiency of DOX with minimal cytotoxicity

For imaging cancer cells, nanotubes were conjugated with a fluorescent dye, fluorescein isothiocyanate (FITC). FITC is a protein labeling reagent useful for fluorescence imaging [18]. FITC was covalently tagged through the side chain amine of lysine present in the polypeptide (Additional file 1: Figure S3a, b). F/P ratio obtained for conjugate was 0.92. To evaluate the cancer cell specificity, HeLa cells and non-tumorigenic keratinocytes (HaCaT cells) were treated with 300 ng/ml of nanotube-FITC conjugate for 4 h. Figure 4 shows the confocal images, which confirm the specific interaction of nanotubes with the cancer cells and also implies that for the time points studied, the nanotube conjugate is located on the cell surface; this suggests that nanotubes can be utilized for release of a drug into the intracellular environment (discussed below). The cellular uptake of nanotube-FITC by cells was visualized in absence and presence of the integrin inhibitor peptide: RGDS. This was done in order to verify that the nanotube conjugate interacts with the cell surface through integrins. Free FITC was used as control for cellular interaction via rapid diffusion (Additional file 1: Figure S4). Upon treatment of cells with nanotube-FITC conjugate at a concentration of 300 ng/ml, no significant internalization of nanotube-FITC conjugate is observed. The nanotube-FITC is mainly distributed over the cell surface of the HeLa cells, whereas non-tumorigenic cells show an overall background signal with no surface binding. As seen in Fig. 5, while HaCaT cells does show minimal uptake of nanotube-FITC conjugate, similar distribution of the conjugate is seen both in presence and absence of the inhibitor.

To evaluate competitive displacement of the nanotubes by the RGD inhibitor, cells were treated for 1 h with 25 µM of inhibitor after 4 h of incubation of cells with nanotube-FITC. Figure 5 shows prominent loss of nanotube-FITC intensity after treatment with the inhibitor. On the other hand, free FITC does not show a significant decrease in its internalization profile in the presence or absence of the inhibitor in HeLa cells (Additional file 1: Figure S4). This demonstrates the specificity of nanotube-FITC interactions with integrin receptors on the cancer cell surface and underscores the potential of the nanotubes in conjugation with fluorescent dyes as a potential tool for imaging cancer cells.

To evaluate the fate of nanotube after binding to the cell surface, we treated HeLa cells for a longer period of time with the nanotube-FITC conjugate. Additional file 1: Figure S3c shows the presence of FITC signal within cellular environment at 8 h, revealing the internalization of FITC tagged nanotubes in cytoplasm. Once internalized, the reducing environment in the cell helps in dissembling the nanotubes, which provides a natural degradation pathway for the nanotubes. The disassembly of the nanotubes in vitro upon addition of a reducing agent has been shown in our earlier studies [15]. This implies that the redox controlled self-assembling nanotubes can be utilized as an efficient drug delivery system.

### Nanotube as a drug carrier: nanotube-DOX

For targeted drug delivery, the presence of multiple RGD units on the nanotubes was employed. Doxorubicin (DOX) was used to measure drug release from nanotubes under pH and redox stimuli. DOX is known to exhibit its antitumor ability by interacting with the cellular DNA in nuclei of cancer cells leading to inhibition of transcription [19]. The hIGFBP-2_249–289_ nanotube constituting a combination of polar and non-polar amino acids along the frame of tubular surfaces makes it an efficient nanocarrier for DOX via electrostatic and hydrophobic interactions. DOX has two possibilities for getting entrapped, either on the exterior of the tube or in the interior (hollow space) of the tube. Zeta potential was used as a measure to probe the electrostatic interaction between DOX and the nanotubes and is shown in Additional file 1: Figure S5a–c. The zeta potential decreases from −21.4 to −12.2 mV for the DOX loaded nanotube, which can be attributed to a rise from the electrostatic attachment of DOX to the nanotube (Additional file 1: Figure S5c). An efficient loading is evident from the characteristic spectral signature seen from UV–Vis spectra in the region 490–500 nm after loading the drug (Additional file 1: Figure S5d). Nanotube-DOX was drop casted on a glass surface and allowed to dry, followed by diffusion limited spreading and the confocal imaging, which confirmed loading of the drug in the interior and exterior of the nanotubes (Additional file 1: Figure S5e). Additional file 1: Figure S5f shows the transmission electron microscopic image for the same nanotube-DOX sample when drop casted on carbon grid.

The efficiency of drug loading was estimated by UV–Vis spectroscopy. The drug loading content (LC) and drug encapsulation efficiency (EE) were estimated (as described in Materials and Methods), which are important parameters that characterize the performance of anti-cancer drug delivery system [20]. The nanotube-DOX had a LC of 55% and EE of 25%.

### Cytotoxicity

In-vitro cytotoxicity of the nanotubes was measured for model cell lines: HeLa, MDAMB231 (Fig. 5) and the non-tumorigenic keratinocytes (HaCaT cells; Additional file 1: Figure S6). Cytotoxicity evaluation was done using MTT assay. First, we evaluated the biocompatibility of the nanotube alone in both the cell lines. As shown in Additional file 1: Figure S6, hIGFBP-2_249–289_ nanotube treatment did not cause any appreciable cytotoxicity after 48 h, suggesting biocompatibility with these cell lines. On the other hand, treatment with 5.5 μM of free DOX for 48 h could kill almost 70% of HeLa and 65% of MDAMB231 cells. In HeLa, the IC_50_ for nanotube-DOX is 0.69 µM and for free DOX is 0.78 µM. In the case of MDAMB231, the IC_50_ was 3.45 µM and free DOX is 3.97 µM. This implies that the nanotube-DOX does not affect the efficiency of free DOX encapsulated within and it is delivering the drug with minimal cytotoxicity.

### Quantification of cellular uptake of DOX

**Fig. 6:**
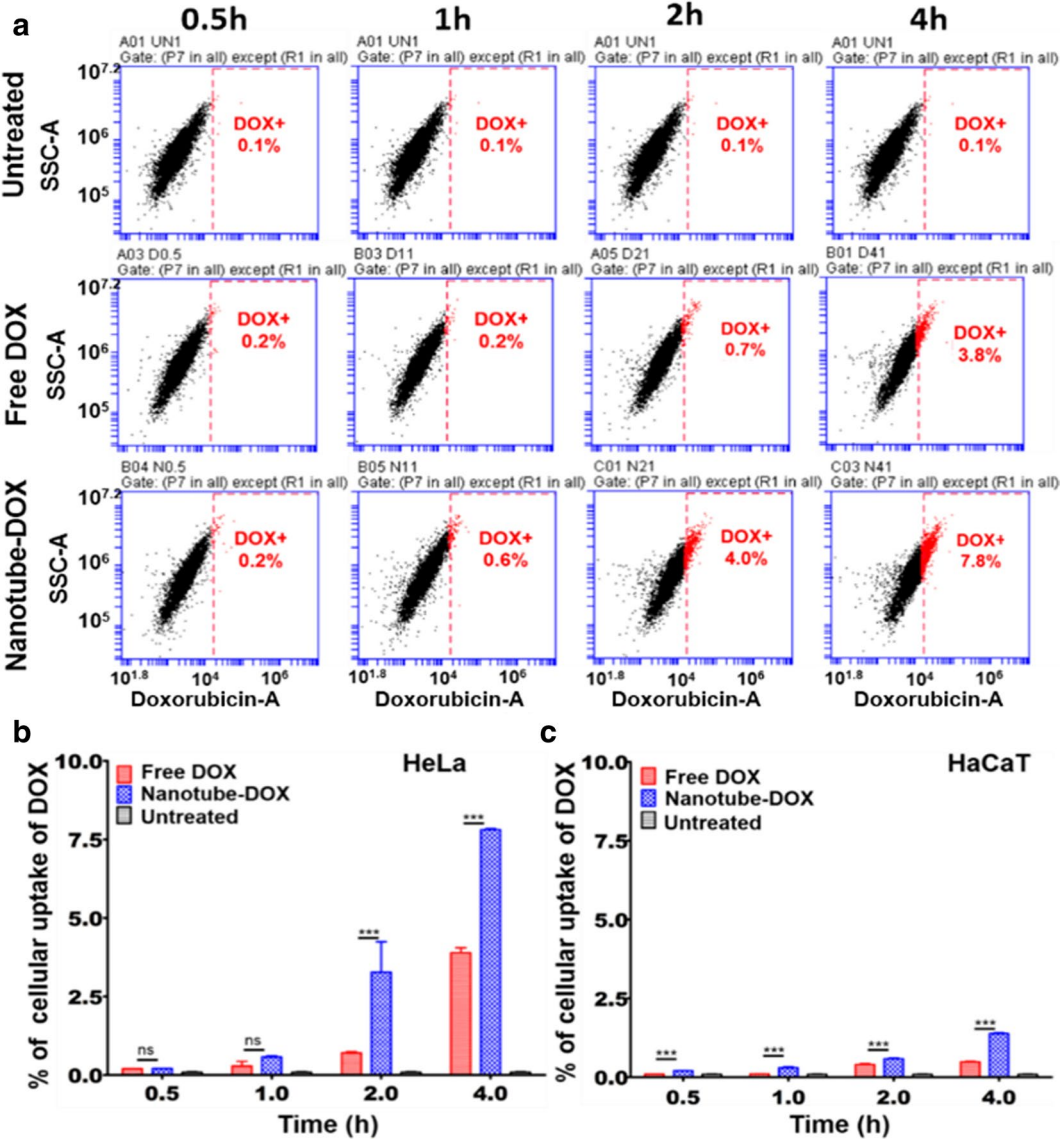
Cellular uptake of DOX. **a** Percentage of cell population positive for DOX internalized at different time points in HeLa cells. **b**, **c** Histogram plots comparing percentage of cell population positive for DOX fluorescence at different time points in HeLa and HaCaT cells, respectively. Percentage obtained for treated culture with nanotube-DOX, free DOX and untreated cells (control)

Cellular uptake of drug was examined at initial time points to evaluate the efficacy of nanotube-DOX over free-DOX and the investigation was done in HeLa and normal keratinocytes (HaCaT). Figure 6 shows quantitative data from the fluorescence assisted cell sorting (FACS) experiment depicting the percentage of HeLa and HaCaT cells, which had taken up the DOX from the growth medium treated with nanotube-DOX and free-DOX separately. The intracellular drug release characteristics of nanotube-DOX and free DOX were compared at concentration of 0.69 µM for each. We quantified the uptake of DOX in terms of percentage population. Early time points: 0.5, 1, 2 and 4 h were chosen to differentiate the targeting and release kinetics of DOX loaded onto the nanotube when compared to free DOX. As shown below in Fig. 6a and b, significant uptake of DOX is observed from 2 h onwards in HeLa cells in the nanotube-DOX system (Fig. 6b) and the uptake of DOX is seen minimal in the normal cells at 2 h. From 2 to 4 h, increase in DOX uptake for nanotube-DOX is more than twofold compared to free DOX in HeLa cells. This indicates an efficient release of DOX from the nanotube attributed to the nanotube interaction with cell surface via integrins.

### Visualization of cellular uptake of DOX

**Fig. 7:**
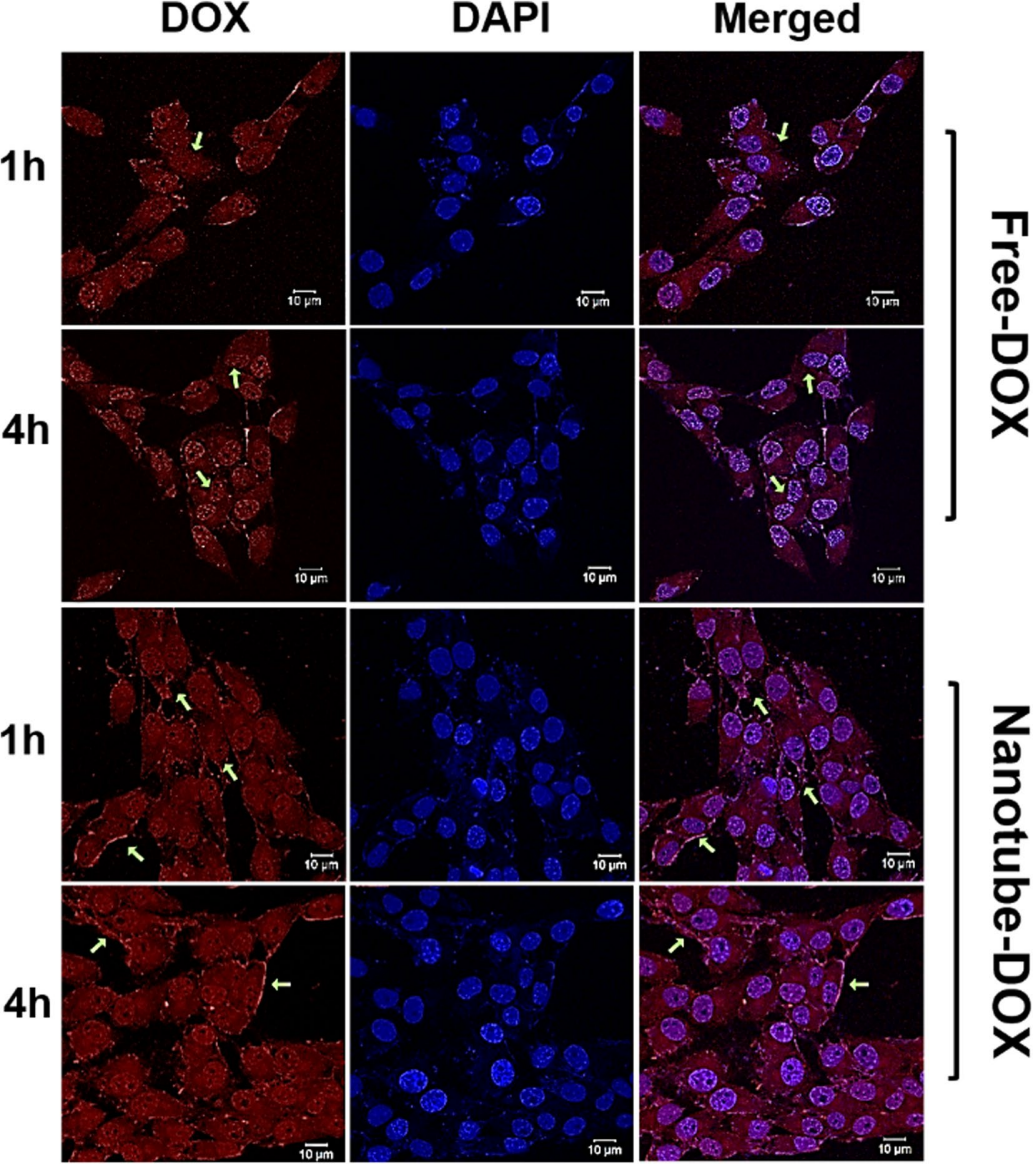
Confocal images for cellular uptake. Visualization of Cellular uptake of DOX by HeLa cells from the treated medium with nanotube-DOX and free DOX at 1 and 4 h

To visualize the drug uptake in HeLa cells, confocal images were acquired after treating the cells with free DOX and DOX loaded nanotubes at the same time points using 0.69 µM each. Figure 7 depicts the internalization of drug upon treatment with free DOX and nanotube-DOX at two-time points 1 and 4 h. In the case of free DOX, major accumulation is seen within nuclei which may be attributed to the rapid diffusion of drug molecules through the cell. Whereas the nanotube-DOX exhibits significant accumulation of drug at the cell membrane and cytoplasm, resulting from nanotube interaction with integrins at cell surface.

## Discussion and conclusions

Nano drug delivery systems are considered as one of the most prospective platforms for cancer therapy because of their important physicochemical properties, including increased drug accumulation in solid tumors by the enhanced permeability and retention (EPR) effect and reduced side effects [21]. In recent years, RGD based targeted delivery systems have come into focus due to their specificity and efficacy [22–28]. For instance, multivalent RGD based peptide nanoparticles have been proposed [22]. A self-assembled multivalent RGD-peptide array has been demonstrated for integrin binding [23]. Functional self-assembling RGD containing peptide nanofiber hydrogel was designed for nerve generation [24]. Disulfide based multifunctional RGD containing conjugate was introduced for targeted theranostic drug delivery [29]. Disulphide connectivity is known to be stable in blood pool but is efficiently cleaved by cellular thiols, including Glutathione and Thioredoxin [29].

The present study introduces the first example of a water soluble self-assembling nanotube formed by a polypeptide fragment from a human protein, which can effectively target cancer cells providing a platform for both imaging of cells and delivery design of suitable therapeutics. The nanotube is formed via redox controlled self-assembly and the array of multiple RGD motifs present on the nanotube makes it suitable for targeting cancer cells via the integrin pathway. This was verified by the induction of integrin mediated pFAK signaling and cellular uptake in the presence and absence of an integrin inhibitor.

The polypeptide fragment is derived from a natural source i.e. polypeptide fragment of human insulin-like growth factor binding protein-2. It therefore exhibits excellent biocompatibility which is reflected in the in vitro cytotoxicity evaluation in cancer cells and non-tumorigenic cells. The intermolecular disulfide formation drives self-assembly of the polypeptide into nanotubular forms providing a stable framework. The nanotubes are stable over a wide range of temperature and pH exhibiting protease-resistance due to Cys281 mutation, which is known to be one of the proteolytic sites in human IGFBP-2 [30]. In addition, the polypeptide hIGFBP-2_249–289_, being part of C-terminal domain of full length IGFBP-2 lacks major proteolytic sites [30, 31] and hence is expected to be unaffected by proteases.

Notably, no additional modification such PEGylation is needed, which is commonly used for providing biocompatibility to various nanoparticles [32] and physical rigidity to the nanoplatforms [33]. Our experiments reveal the potential of this system for targeted therapeutics that includes drug delivery and imaging. In vitro drug release profiles confirm its ability to effectively deliver the cargo. Disulfide connectivity makes to redox responsive to the cellular GSH and lysosomal environment, which again renders the nanoplatform as a biocompatible redox responsive system suitable for targeted therapy. The cellular uptake of nanotube-FITC at 8 h reveals the presence of the nanotube-FITC signal in intracellular environment which can be a result of redox response of the nanotube.

**Fig. 8:**
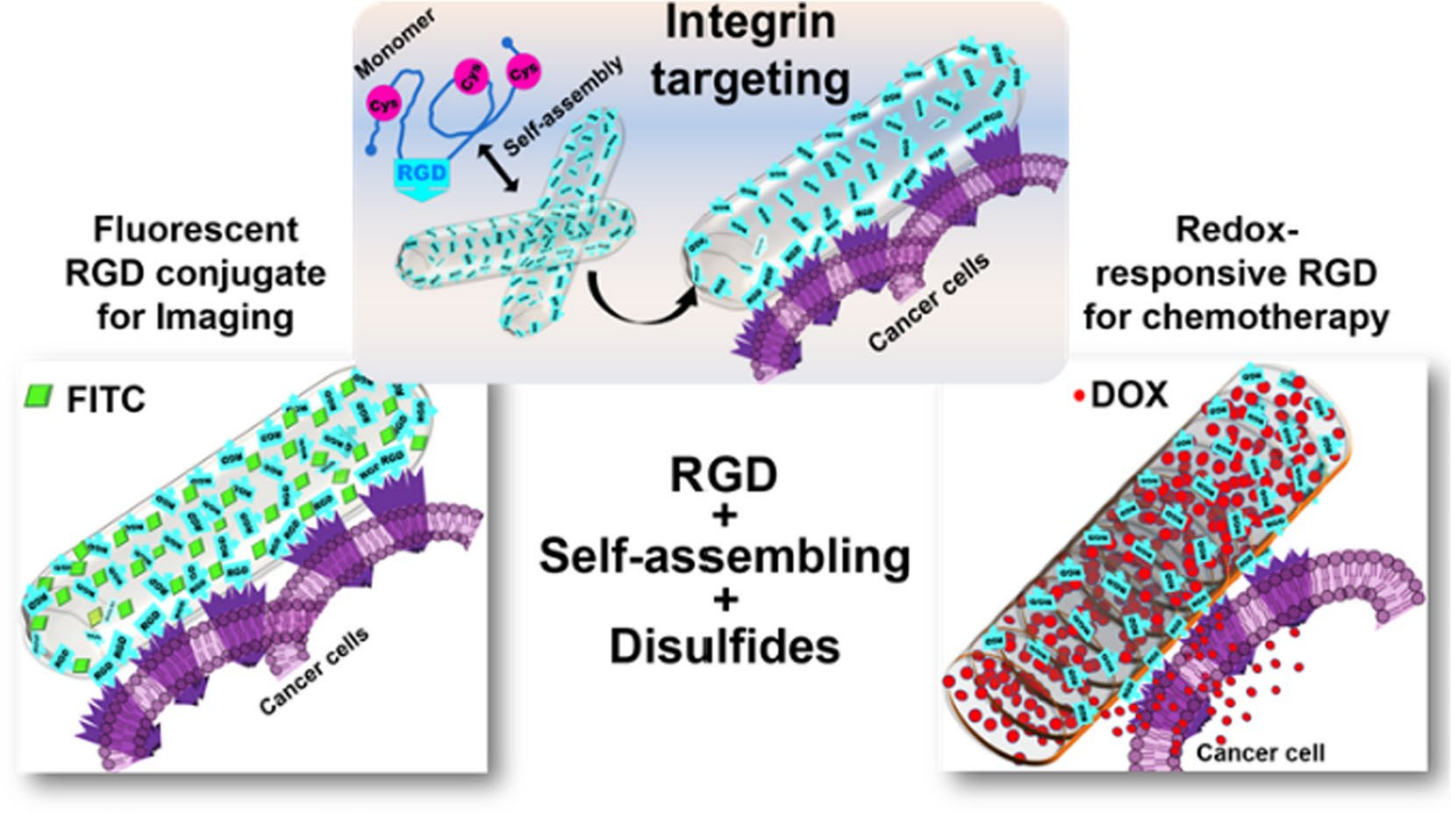
Schematic demonstrating the summary, RGD containing peptide based nanotube is proven to be successful in targeting integrins and shown to be useful for the targeted imaging and therapy in cancer cells

In summary, we provide evidence for RGD based natural polypeptide nanotubes as effective delivery system for imaging and treatment with cytotoxic drugs. The targeted delivery has the potential to minimize the ill effects of the cytotoxic drugs on normal surrounding cells thus alleviating the side effects during the treatment of cancer. This is depicted below schematically (Fig. 8).

## Experimental methods

### Preparation of the protein nanotubes

#### Expression and purification of monomer

To express the monomeric form of the protein, the pGEX-6p2-IGFBP-2_249–289_ (C281) plasmid10 was used for transformation of BL21 (DE3) pLysE *E. coli* cells. The glutathione-S-transferase (GST) Gene Fusion system was used for the over expression and purification of the construct [16]. The amino acid sequence of the polypeptide is:

“GPLGSPGIRGSCVNPNTGKLIQGAPTIRGDPECHLFYNEQQEACGVHTQRMT”.

The underlined first 11 N-terminal residues correspond to additional amino acids introduced by the gene fusion system as described previously [16]. For bacterial culture, 10 ml of Luria–Bertani (LB) (Hi-media, M575) medium containing 100 µg/ml Ampicillin (Himedia, CMS645) was inoculated with a transformed colony and grown overnight at 37 °C. Later, cells were regrown to mid-log phase (OD_600_ nm ~ 0.6) at 37 °C with 100-fold dilution from overnight primary culture in fresh 100 µg/ml Ampicillin containing LB medium. Overexpression of the fusion protein was achieved by inducing the cells with 1 mM isopropyl β-d-thiogalactoside (IPTG, Calbiochem, 420322, India) for 6 h at 30 °C. Cells were harvested by centrifugation at 6000 rpm for 20 min, followed by cell lysis in phosphate buffered saline (PBS), pH 7.5, 1 mM PMSF (phenylmethylsulfonyl fluoride, Himedia, cat no. India), on ice by sonication in six steps, 20 s cycles each, with an intervening period of 2.0 min. Cell lysate was centrifuged (twice) at 30,000*g* for 45 min at 4 °C to separate insoluble cell debris and the soluble fraction containing the protein. Soluble fraction was loaded on 50% slurry of pre-equilibrated glutathione-sepharose beads (Novagen, 70541, India) with PBS at 4 °C, for 3 h on nutator. The fusion protein-bound to the affinity beads was collected by centrifugation at 4000 rpm for 5 min and washed three times with 10 bed volumes of PBS (pH 7.5, 50 mM phosphate, 50 mM NaCl). It was further washed three times with 10 bed volumes of high (25 mM HEPES, 0.05% NaN3, 0.5 M NaCl, and 0.1% Triton X-100, pH 7.5) and low (25 mM HEPES, 0.05% NaN3, 0.1 M NaCl, and 0.1% TritonX-100, pH 7.5) salts. This was followed by washing twice with cleavage buffer (1.5 M NaCl, 0.5 M Tris–HCl, pH 7.5) to remove impurities and non-specifically bound protein. On column cleavage was performed by pre-scission protease HRV-3C Protease (Human rhinovirus 3C protease cleaves at Q-G bonds, Millipore, 71493-3, India). 10 µl (20 units) of HRV-3C Protease was added for each millilitre of glutathione-sepharose bed volume, in addition with 490 µl of cleavage buffer. It was then nutated at 4 °C for 16 h. Cleaved protein was eluted by centrifuging at 4000 rpm, 5 min and further subjected to exchange with PBS buffer (pH 7.4). Purity of purified protein was confirmed by loading it on SDS-PAGE. The monomeric form of the protein was then allowed to undergo oligomerization in PBS to form self-assembled nanotubes. No external agent was necessary for initiating the oligomerization. Formation of the nanotubes was monitored by SDS-PAGE, NMR spectroscopy, TEM and Dynamic Light Scattering (DLS).

### Characterization of nanotubes by NMR spectroscopy

The monomer (hIGFBP-2_249–289_ (Cys281)) and the nanotubes formed were characterized by NMR spectroscopy. The incorporation of NMR active isotopes i.e. ^13^C and ^15^N into the protein was achieved by growing the transformed BL21 (DE3) pLysE *E. coli* cells in a minimal (M9) medium containing ^13^C_6_-glucose [34] and/or ^15^NH_4_Cl [35] as the sole sources of anabolic carbon and nitrogen. Bacterial culture and purification of the proteins were carried out using previously described protocol. Protein estimation was done using Lowry’s method [36].

### NMR experiments

About 0.8 mM purified protein was dissolved in PBS and 5% ^2^H_2_O (for locking). All NMR data were recorded at 298 K on a Bruker Avance III 800 MHz NMR spectrometer equipped with a cryogenically cooled triple resonance probe. Two-dimensional (2D) [^13^C, ^1^H] HSQC was acquired with 8 transients and 256 complex points with a measurement time of 40 min each and 2D [^15^N, ^1^H] HSQC spectra were acquired with 2 transients and 256 complex points, with a measurement time of 10 min each. The 2D [^15^N, ^1^H] HSQCs experiment was used for time dependent monitoring of oligomerization of self-assembling hIGFBP-2_249–289_ (Cys281) for 3 days and 2D [^13^C, ^1^H] HSQCs were acquired to monitor the oxidation of Cysteine during self-assembly as a function of time for 8 days.

### Assessment of oligomerization by SDS-PAGE

Self-assembly of hIGFBP-2_249–289_ (Cys281) into nanotubular structures in the initial stages was monitored by SDS-PAGE. This was carried out in parallel with the 2D [^15^N, ^1^H] HSQC NMR experiment mentioned above. Protein fractions were collected on day 1, day 2 and day 4 and stored at − 20 °C to arrest the oligomerization. Samples for SDS-PAGE were prepared in a buffer containing 240 mM Tris–HCl (pH 6.8), 8% (w/v) SDS, 0.1% Bromophenol blue and 40% (v/v) glycerol. Protein fractions taken in this buffer were heated at 96 °C for 10 min, and then centrifuged briefly. The samples were resolved on 15% SDS–polyacrylamide gels in Tris–glycine electrophoresis buffer (pH 8.3) containing 25 mM Tris base, 250 mM glycine and 0.1% SDS at 100 Volts for 120 min. Coomassie brilliant blue g-250 staining was performed for visualization of oligomeric bands.

### Preparation of nanotube conjugated with FITC

FITC (Sigma, India) was conjugated to nanotubes using the procedure described previously [37]. All steps were performed in dark conditions to avoid photo hydrolysis of FITC. First, a solution containing 1 mg/ml of the protein nanotubes was prepared in borate buffer (0.5 M, pH 8.5). The FITC was added to this solution in the ratio of 1:10 (nanotube: FITC) with continuous stirring. Reaction mixture was shaken well to obtain uniform dissolution of FITC and protein. It was then kept at 4 °C for nutation overnight. The product (nanotube-FITC conjugate) was subjected to dialysis against borate buffer to remove unconjugated FITC using a 1 kDa membrane cut-off eventually exchanging with Milli-Q water. After complete buffer exchange, the final conjugate was lyophilized and quantified. For experimental use, the lyophilized conjugate was dissolved and stored in PBS (50 mM, pH 7.4) with 0.02% NaN_3_ (an antibacterial agent). FITC/Protein (F/P) ratio of the conjugate was calculated according to the equations

$$Molar{\raise0.7ex\hbox{$F$} \!\mathord{\left/ {\vphantom {F P}}\right.\kern-0pt} \!\lower0.7ex\hbox{$P$}} = \frac{MW}{389} \times \frac{{A_{495} /195}}{{\left( {A_{280} - \left( {0.35 \times A_{495} } \right)} \right)/E^{0.1\% } }} = A_{495} \times C$$

where, $$C = \frac{{MW \times E_{280}^{0.1 \% } }}{389 \times 195}.$$

C is a constant value given for a protein, MW is the molecular weight of the protein, 389 Da is the molecular weight of FITC, 195 is the absorption E^0.1 %^ of bound FITC at 490 nm at pH 8.0 (0.35 × A_495_) is the correction factor due to the absorbance of FITC at 280 nm [38], E^0.1 %^ is the absorption at 280 nm of a protein at 1.0 mg/ml.

### Preparation of nanotube-DOX

Self-assembled nanotubes were subjected to exchange with Milli-Q water using a 3 kDa 15 ml Centricon tube (Millipore) spun at 4000 rpm at 4 °C. Stability of the nanotube in pure water was confirmed by TEM. The sample was lyophilized and the dry weight was taken to quantify the yield. Adsorption of Doxorubicin hydrochloride (purchased from Sigma, India) on nanotube was carried out by mixing a sample of DOX prepared in water at a concentration of 2 mM with 100 µM of nanotubes (corresponding to a protein: DOX ratio of 1:20) and the mixture was nutated for 12 h at 4 °C. Unbound DOX was removed by dialysis against pure water using 1 kDa membrane (Millipore). Drug Encapsulation efficiency and drug loading content was calculated as below:

$${\text{Encapsulation efficiency }}\left( {\text{EE}} \right) \, \left( \% \right) \, = \, \left( {{\text{W}}_{0} - {\text{W}}_{\text{d}} /{\text{ W}}_{0} } \right) \, \times { 1}00$$

$${\text{Loading content }}\left( {\text{LC}} \right) \, \left( \% \right) \, = \, \left( {{\text{W}}_{0} - {\text{W}}_{\text{d}} /{\text{ W}}_{\text{NT}} } \right) \, \times { 1}00$$

W_0_ represents the initial amount of drug fed, W_d_ represents amount of drug released in dialysate, W_NT_ represents weight of nanotubes used for drug loading.

### Transmission electron microscopy (TEM)

For acquiring TEM images, the nanotubes were prepared at room temperature (25 °C) by allowing self-assembly under redox control i.e. having no reducing agent present in PBS buffer. The TEM studies were recorded under 200 kV on a Technai F-30 TEM instrument equipped with a cryoprobe. Drop casting method was used and samples were stained with Uranyl acetate (0.5%), then air dried for 1 h and desiccated for overnight on copper grids of 200 mesh.

### Fluorescence spectroscopy

Fluorescence spectra were acquired on a Perkin Elmer Fluorescence spectrometer. Spectra were recorded for protein samples prepared in 10 mM Tris buffer at pH 7.4 and data were recorded using a 1.0 cm path length quartz cuvette. An excitation wavelength of 280 nm (tyrosine excitation) was used having slit width 2.5 nm and emission spectra were collected from 300 to 550 nm with λ_max_ at 340 nm.

### UV–Visible spectroscopy

The UV–Visible spectra were recorded for nanotube-DOX and nanotube-FITC conjugate on a Shimadzu UV-1800 UV–Vis spectrophotometer with slit width of 1 nm using a quartz cuvette having a path length of 1 cm with a wavelength range of 200–800 nm.

### Dynamic light scattering (DLS)

The Nano tubular size distribution was measured on Zetasizer (Malvern, Southborough, MA, USA). The changes in the surface charges of free nanotube and nanotube-DOX were probed via the zeta potential using Nano ZS (Malvern, Southborough, MA, USA) [39].

### Atomic Force microscopy (AFM)

AFM experiments were performed on a NX-10 AFM (Park systems) system in the non-contact mode. The protein sample was dropped onto freshly cleaved mica and incubated for 20 min. After rinsing with Milli-Q water twice, the sample was dried in a desiccator. The Al back-coated Si probe (ACTA, AppNano Inc, USA) had a resonance frequency of 300 kHz and nominal spring constant of 40 Nm^−1^. The tip radius was < 10 nm. Images were obtained at a scan rate of 1 Hz.

### Cell culture

HeLa (Cervical cancer cell line), MDA-MB-231 (Breast cancer cell line) and HaCaT (Human keratinocytes) cells were maintained in growth medium consisting of Dulbecco’s Modified Eagle’s Medium (DMEM, Gibco, India) with high glucose, supplemented with 10% FBS and 1% penicillin–streptomycin. The cells were grown in 75 mm^2^ flasks (BD Falcon) and passaged every 3 days. All culture reagents were purchased from Thermo fisher scientific. The serum free media was used to eliminate any confounding effects from serum adsorption to the nanotubes. Within the time-period (5 h) of the experiment, no adverse cellular responses were observed from serum deprivation or serum shock after a transfer from serum containing growth media.

### Cytotoxicity assay

Cytotoxicity of nanotube, DOX loaded nanotube and free DOX were assessed in HeLa and MDAMB231 using the MTT ((3-(4,5-dimethylthiazol-2-yl)-2,5-diphenyltetrazolium bromide) assay. About 5 × 103 cells were seeded per well of a 96-well plate and allowed to attach during incubation for 24 h in a humidified incubator at 37 °C. Cells were treated with different concentrations of nanotubes, free DOX or DOX loaded nanotubes ranging from 0.2 to 6.4 μg/ml. 4 h before termination of the assay, and MTT was added. Mitochondria of viable cells oxidize MTT to produce purple Formazan crystals. Formazan crystals were dissolved in 100 μl of DMSO and incubated for 15 min with shaking at RT, protected from light. The absorbance of each well was measured at 570 nm using a microplate reader (Synergy HT, BioTek Instrument Inc., Winooski VT). Absorbance was converted to the percent cell viability and IC_50_ concentrations calculated as the concentration that caused 50% inhibition of cell growth.

### Western blot analysis

Cells were maintained as mentioned above in 65 mm dishes. HeLa cells were serum starved for 24 h to remove the effect of serum on cells. Cells were treated with nanotubes for 1 h in serum-free DMEM. An additional set of cells was treated for 1 h with nanotubes without the integrin pathway inhibitor (RGDS) under the same conditions. For control treatments, cells were pretreated with 25 µM RGDS peptide for 1 h. Later, cells were washed once with DPBS (Dulbecco’s phosphate-buffered saline) and lysed in detergent lysis buffer (150 mM NaCl (Sigma Aldrich, USA), 0.1% SDS (Calbiochem, Germany), 0.5% NP40 (Amresco, USA), 1 mM EDTA (pH 8) and protease inhibitor cocktail, PIC (Calbiochem) with gentle vortexing at 4 °C for 20 min and centrifuged at 10,000 × g for 10 min. The soluble fraction was separated and, protein estimation was done by Bradford assay. 30 µg of total protein was dissolved in SDS sample buffer (240 mM Tris–HCl (pH 6.8), 8% (w/v) SDS, 0.1% bromophenol blue and 40% (v/v) glycerol), heated at 96 °C for 10 min and centrifuged briefly. Samples were resolved on 12.5% SDS–polyacrylamide gels in Tris–glycine electrophoresis buffer (pH 8.3) containing 25 mM Tris base, 250 mM glycine and 0.1% SDS at 100 Volts for 2 h. Proteins were then transferred from the gel onto a PVDF membrane (Immobilon-P, Millipore) in 25 mM Tris base, 250 mM glycine and 20% methanol using a BioRad transfer apparatus (BioRad Laboratories, USA). Membranes were blocked with 5% non-fat dry milk solution prepared in Tris buffered-saline, pH 7.4 containing 0.1% Tween 20 (TBST) for 1 h at room temperature and then probed with the respective primary antibody at their prescribed dilutions in 2% BSA in TBST (TBT) overnight at 4 °C. The following antibodies were used for Western blotting: Phospho FAK Tyr 397 (#3283, Cell signaling), Total FAK (#3285, Cell signaling) and β-Actin (#A5441, Sigma-Aldrich, USA). After primary antibody incubation, membranes were washed three times (15 min each) with TBST and incubated with HRP-conjugated secondary antibodies (Santa Cruz, USA) for 1 h at room temperature followed by washing three times (15 min each) with TBST. Enhanced chemiluminiscence substrate (#786-00, femto LUCENTTM PLUS-HRP, G-biosciences) or SuperSignal West Femto substrate (Pierce Protein Research Products, Thermo Scientific, USA) was added followed by exposing the membrane to X-ray film, development and fixation. Developer and fixer were purchased from Eastman Kodak Company, USA.

### Confocal image acquisition and analysis

For microscopic analysis, cells were grown on sterile coverslips with all treatments performed in DMEM without serum for 1 or 4 h. Treatment was terminated by removing medium and cells washing cells twice with PBS followed by nuclear staining with DAPI (4′,6-diamidino-2-phenylindole) for 10 min at room temperature. Cells were then washed with DPBS and excess buffer was removed. Coverslips were then treated with Antifade (Thermo Fisher Scientific India Private Ltd.) and sealed to a sterile glass slide and analysed by confocal microscopy (LSM 510 Meta equipped with an Airyscan module, Zeiss, GmbH, Germany).

### FACS analysis

To quantify cellular uptake, we have used FACS based approach. 80,000 cells were seeded for each sample. Cells were first gated for auto fluorescence, 0.1–0.5% population was selected to ensure no loss of data points of experimental samples. During acquisition data, 20,000 events were recorded and analysed. Cells positive for DOX fluorescence were plotted as the bar graph. The cells were treated with free DOX and nanotube-DOX for 0.5, 1, 2 and 4 h using concentrations corresponding to their IC_50_ values. The cells were washed with DPBS to remove the unassimilated DOX and then trypsinized. The cell suspension was then centrifuged, and the pellet was re-suspended in 300 μl of DPBS with 0.1% of FBS. The DOX uptake was estimated by quantifying the cell populations using FACS employing Cell Quest software (using a FACS Calibur instrument, BD Biosciences, San Jose CA).

### Statistical analysis

All statistical analyses were performed by using the GraphPad 5.03 statistical software and all data were plotted as mean ± SD (standard deviation). Comparisons among multiple groups were assessed by two-way ANOVA, and *p value < 0.05, **p value < 0.01, ***p value < 0.001 were considered statistically significant.

## Additional file

**Additional file 1.** Supporting Information.
