# Supplementary material for: An ultra-stable redox-controlled self-assembling polypeptide nanotube for targeted imaging and therapy in cancer
Source: J Nanobiotechnology. 2018 Dec 8;16:101. doi: 10.1186/s12951-018-0427-1 (PMC6286583; doi:10.1186/s12951-018-0427-1)

Additional Information

**An Ultra-Stable Redox Controlled Self-Assembling Polypeptide Nanotube for Targeted Imaging and Therapy in Cancer**

*Gitanjali Asampille^ab^, Brijesh Kumar Verma,^c^ Monalisa Swain^abe^†, Abhijith Shettar^cf^†, Steven A. Rosenzweig^d^, Paturu Kondaiah^c*^ and Hanudatta S. Atreya^a^**

^a^NMR Research Centre, Indian Institute of Science, Bangalore-560012

^b^Solid State and Structural Chemistry Unit, Indian Institute of Science, Bangalore-560012

^c^Molecular Reproduction, Development and Genetics, Indian Institute of Science, Bangalore-560012

^d^Department of Cell and Molecular Pharmacology & Experimental Therapeutics, Medical University of South Carolina, SC 29425

**Figure S1**. Monitoring oligomerization as a function of time using 2D [^15^N, ^1^H] HSQC NMR experiment on day 1, day 2 and day 4. HSQC spectra (Top), expanded region representing the new species arising as a function of time (bottom).


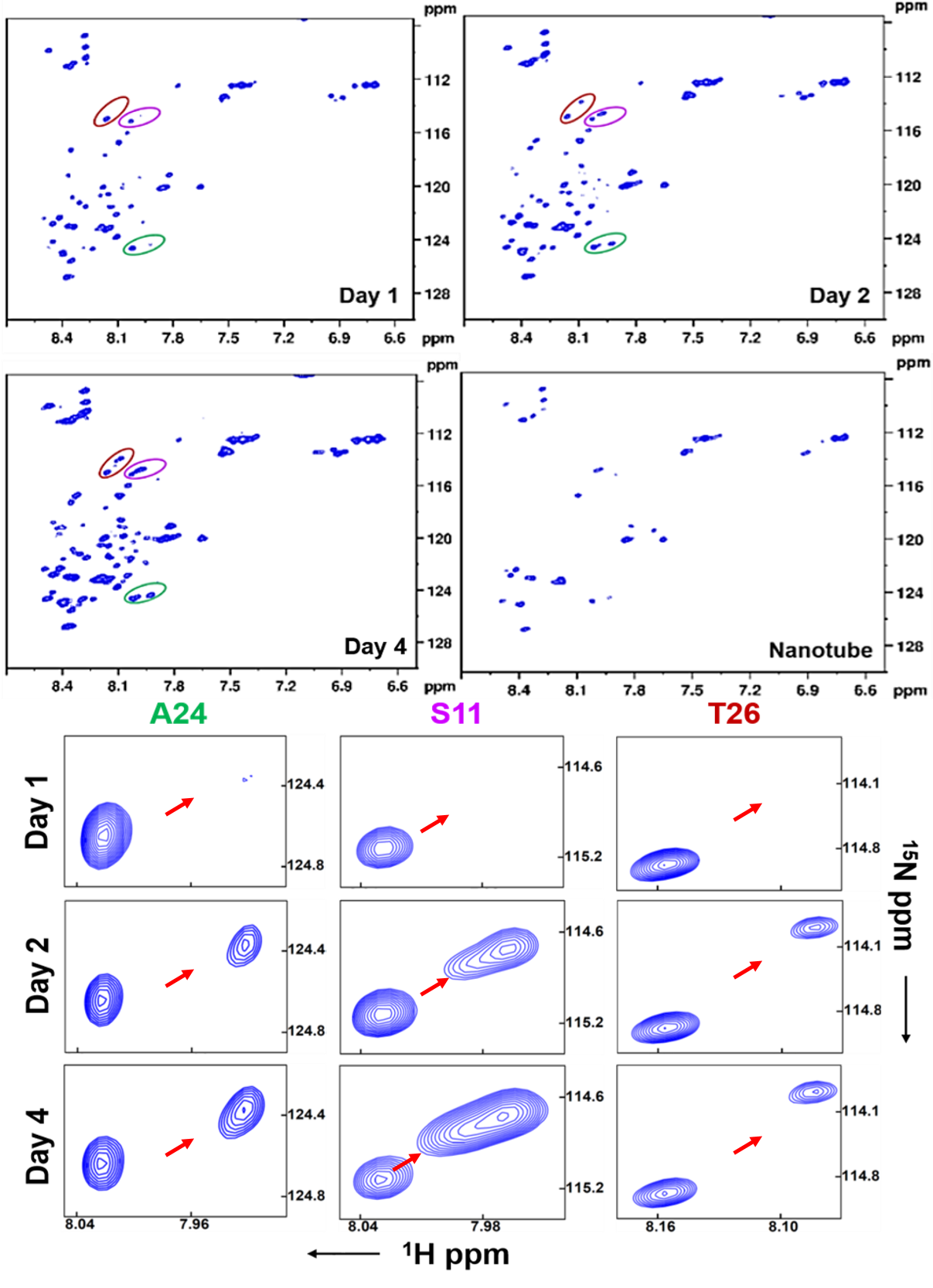


**Figure S2**. Size distribution and Zeta potential profiles (a) Dynamic light scattering profile of nanotubes, (b) Zeta potential for the nanotube

**Dynamic Light Scattering**

We used dynamic light scattering instrument with fixed detection angle of 90° to investigate the size distribution of hydrodynamic radii of mature nanotubes. We observe bimodal profile for nanotube owing to its non-spherical form with large aspect ratio^39^, the intensity distribution (expressed as the percentage of the total scattered light intensity) exhibit major size distribution peak around 512 nm and small size peak around 70 nm (Figure S2a).


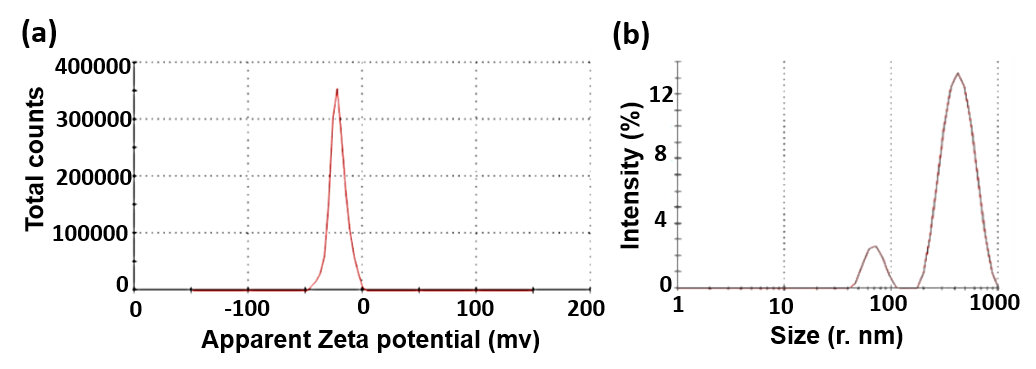


**Figure S3**: Nanotube-FITC preparation and characterization (a) UV -Vis spectra for free FITC and Nanotubes Conjugated with FITC, (b) Upon UV excitation FITC tagged nanotubular intermediate, (c) Cellular uptake of nanotube-FITC in HeLa cells at 8 h.


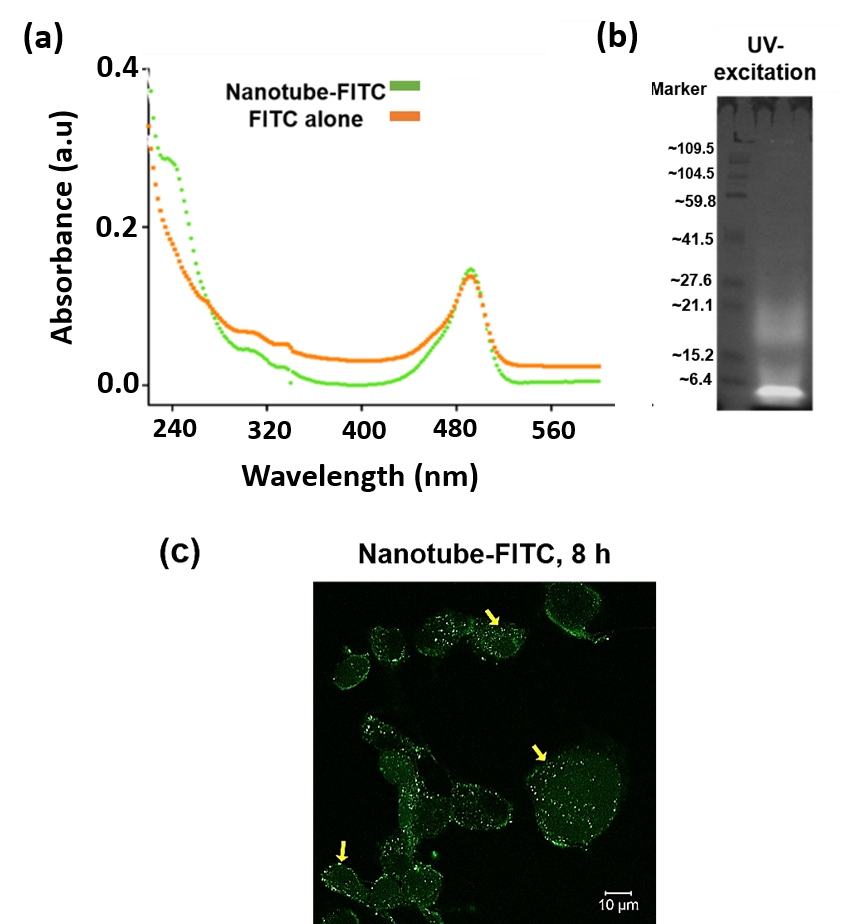


**Figure S4**: Free-FITC interaction with HeLa cells, free FITC rapidly diffused in the cells in absence of the inhibitor (left column) and cell uptake following 1 h treatment of the integrin inhibitor given after 4 h of incubation of the conjugate with the cells (right column). Cell nuclei are stained with DAPI (blue color) and shown are the overlay of images (merged) acquired using FITC and DAPI channel.


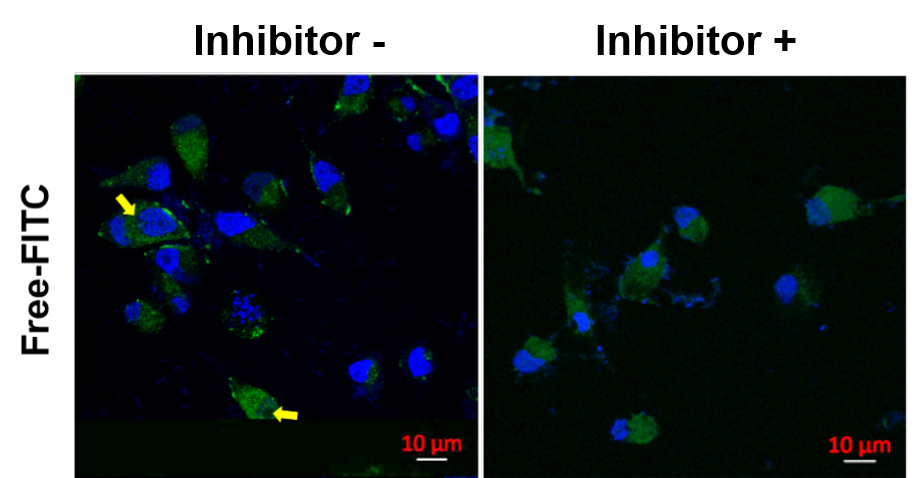

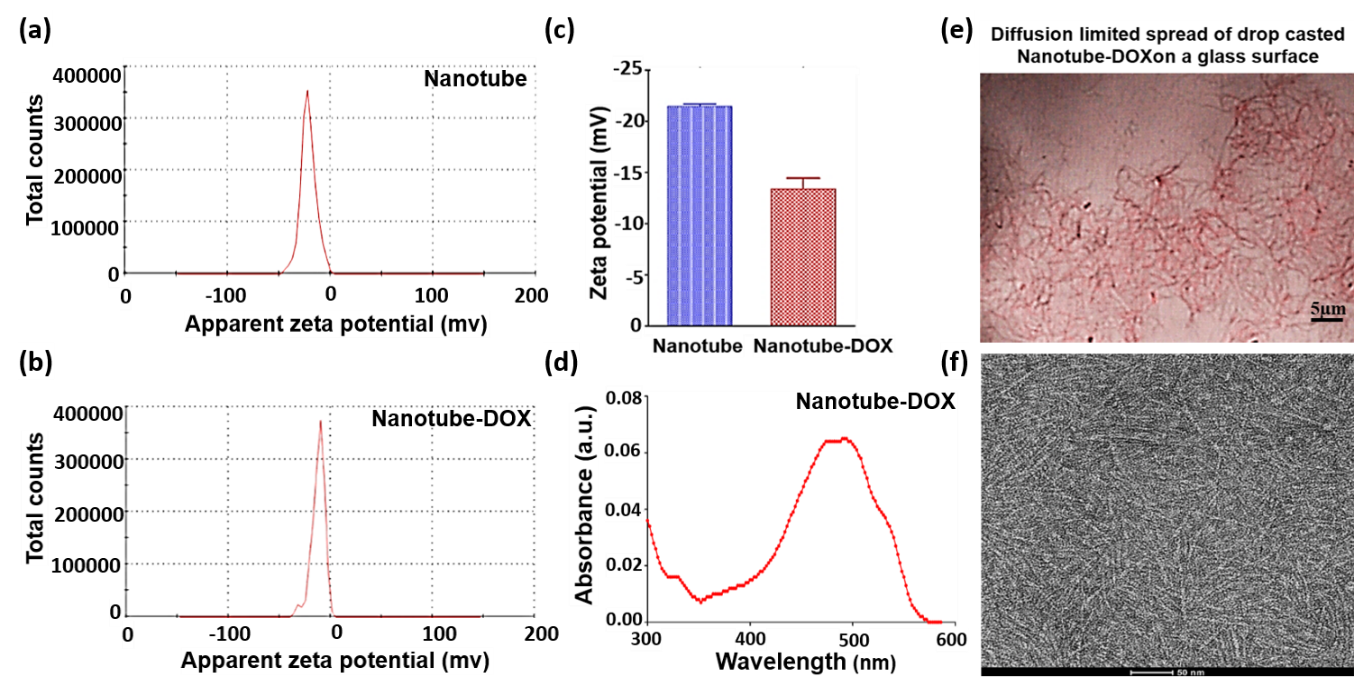


**Figure S5.** Nanotube-DOX preparation and characterization, (a) Zeta potential profiles for nanotube and (b) nanotube-DOX system, (c) Histogram comparing the zeta potentials both before and drug loading on nanotubes, (d) UV-vis spectrum showing characteristic peak for Doxorubicin loaded on nanotube, (e) Confocal image of Doxorubicin loaded Nanotube coated on a glass surface, (f) TEM image after loading Doxorubicin on nanotubes.


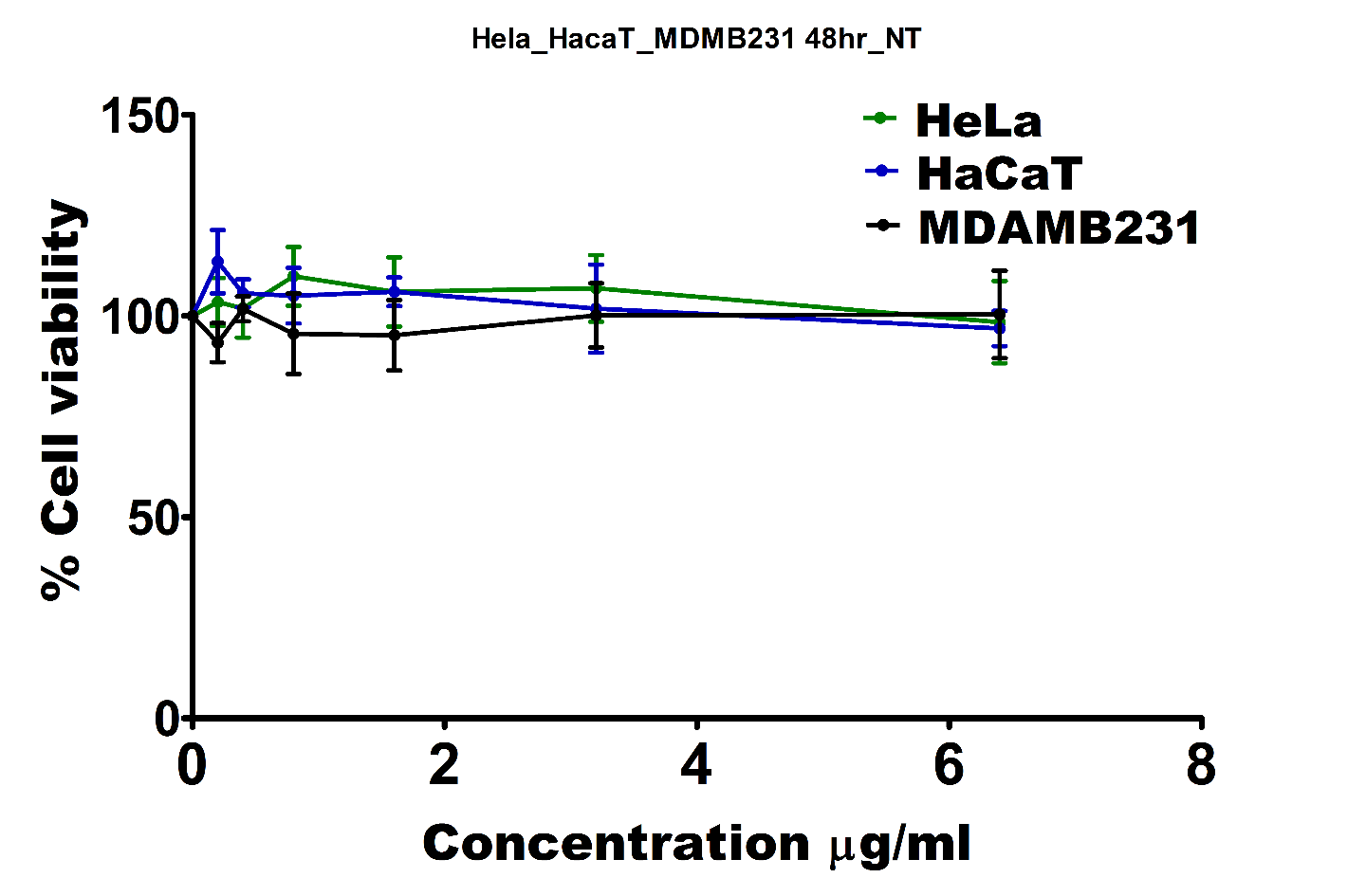


**Figure S6**: Cytotoxicity assay, Comparing cytotoxicity profile of nanotubes obtained in three different cell lines at 48 h.

**Figure S7**: Cellular uptake of DOX via nanotube-DOX, free DOX and untreated (Control) for HaCaT cells. Percentage of cell population positive for internalized DOX fluorescence at different time points.


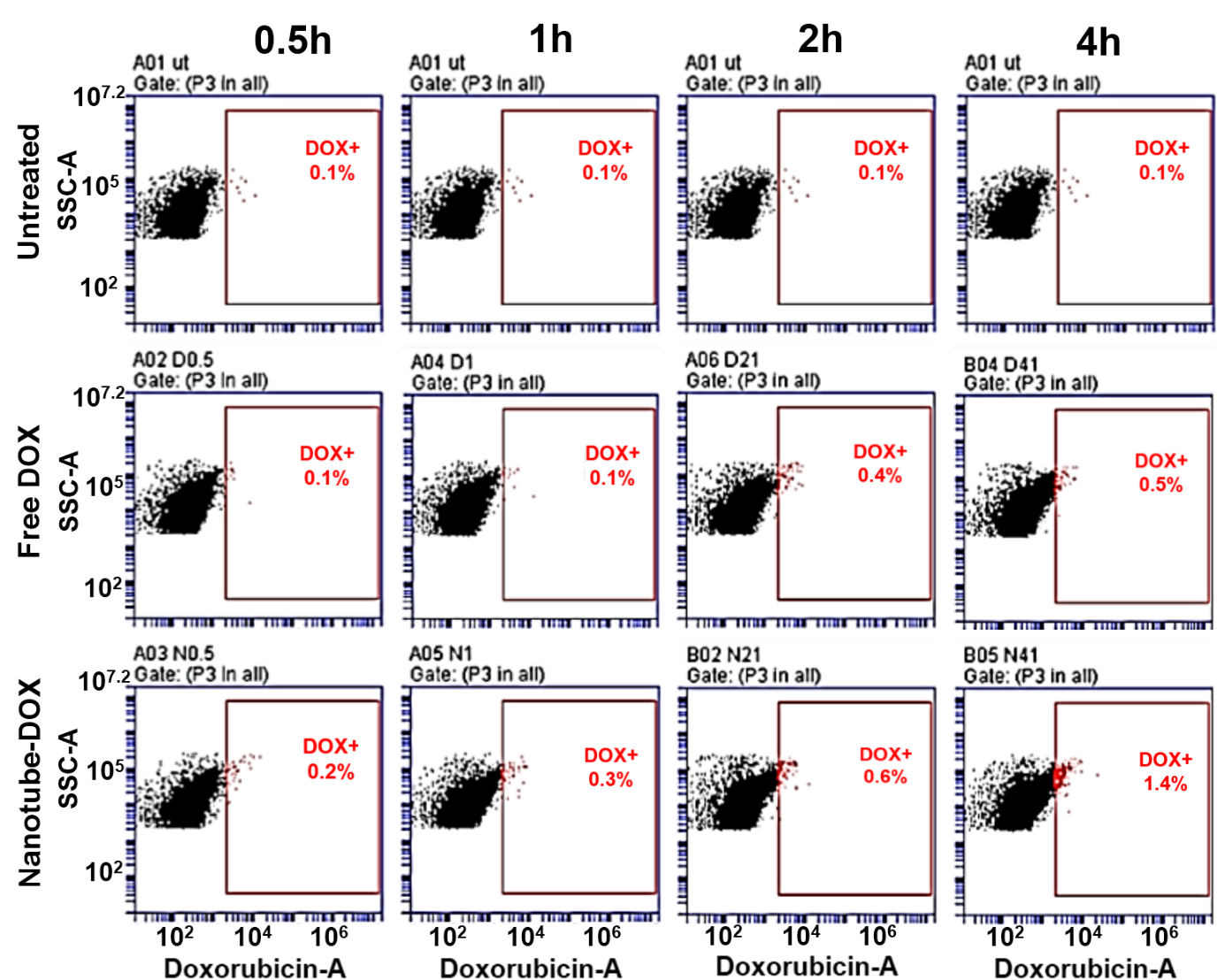

Supplement: Supplementary file 1 — Additional file 1. Supporting Information. [file 12951_2018_427_MOESM1_ESM.docx]
